# Dietary quality and patterns and non-communicable disease risk of an Indian community in KwaZulu-Natal, South Africa

**DOI:** 10.1186/s41043-015-0013-1

**Published:** 2015-08-08

**Authors:** A. Naicker, C. S. Venter, U. E. MacIntyre, S. Ellis

**Affiliations:** 1 Department of Food and Nutrition Consumer Sciences, Durban University of Technology, Kwa-Zulu Natal, South Africa; 2 Centre of Excellence for Nutrition, Faculty of Health Sciences, North-West University, North West Province, South Africa; 3 Department of Human Nutrition, University of Pretoria, Gauteng, South Africa; 4 Statistical Consultation Services, North-West University, North West Province, South Africa

**Keywords:** Diet quality, Diet patterns, Non-communicable diseases

## Abstract

**Background:**

Limited data exist on the South African Indian diet despite their high prevalence of non-communicable diseases. This study attempted to determine the dietary quality and patterns of an Indian population in KwaZulu-Natal with reference to the high prevalence of non-communicable disease

**Methods:**

Two-hundred-and-fifty apparently healthy Indians, aged 35–55 years participated in a cross-sectional study where diet was assessed using a validated quantitative food frequency questionnaire. Mean intakes were compared to the World Health Organization goals. Dietary quality was determined by index construction and dietary patterns by factor analysis.

**Results:**

The mean daily percentage of energy (%E) from n-3 fatty acids (0.24 %E), dietary fibre (18.4 g/day) and fruit and vegetable intakes (229.4 g/day) were below the World Health Organization goals. Total fat (36.1 %E), polyunsaturated fatty acids (11.8 %E), n-6 fatty acids (11 %E) and free sugars (12.5 %E) exceeded the goals. The means for the deficient index reflected a moderate diet quality whereas, the excess index reflected good diet quality. The Pearson partial correlation coefficients between the deficient index and risk markers were weak whilst, the excess index was inversely correlated with waist circumference for the whole sample. Two factors were identified, based on the percentage of fat that contributed to each food group: factor 1 (meat and fish versus legume and cereal pattern), which accounted for added fat through food preparation; and Factor 2 (nuts and seeds versus sugars and visible fat pattern), which accounted for obvious fat. The medians for waist circumference, blood glucose, cholesterol and triglyceride levels showed significant decreasing trends for factor 1 (*p* < 0.05). The medians for blood glucose and cholesterol showed significant decreasing trends for factor 2 (*p* < 0.01).

**Conclusion:**

A shortfall of fruit and vegetable, fibre and n-3 fatty acid intake in the diet is highlighted. When assessing the diet quality and patterns, guidance on the prudent use of added fats may lead to a healthier lifestyle reducing the prevalence of non-communicable diseases.

## Background

Global diet is undergoing an alarming transition: staple foods are becoming more refined and processed; fat and meat intakes are increasing; more processed dairy products and other foods are consumed; and larger numbers of meals are consumed outside the home, making households more reliant on the food industry. Dietary habits have collectively been influenced by urbanisation, economic development, market globalisation and industrialisation where the move from a traditional to a Western-type diet is a characteristic feature [1]. Consequently, this transition has been associated with an increase in the global prevalence of non-communicable diseases (NCDs) [2].

The role of diet in the aetiology of most NCDs is extremely important. A body of literature attests to the fact that diets and specific nutrient deficiencies and excesses influence the development of NCDs and that appropriate dietary changes may reduce the risk of NCDs [2]. Little is known about the South African Indian diet, despite their high incidence of diabetes mellitus [3] and coronary heart disease (CHD) [4]. The first migrant Indians were reported to arrive in South Africa in 1860 to work as sugarcane farm labourers. With equity and social transformation, their lifestyles have changed rapidly for the better, however, their risk for NCDs have reached epidemic proportions [5, 6].

Diet is considered a modifiable risk factor for NCDs [7]. Over recent years dietary assessment has moved away from single nutrients to assessing the whole diet and interrelations between dietary factors in relation to NCDs [8, 9]. Therefore, a number of epidemiologists propose using a dietary pattern approach to investigate food in relation to NCDs [10, 11]. Most studies use either, a priori methods such as diet indices to assess the quality of the diet or a posteriori data-driven methods, such as factor analysis to derive food patterns in an attempt to describe the dietary behaviour of a population [12] and the associated risks thereof.

The primary hypothesis of this study was that dietary quality and patterns are related to risk markers for developing nutrition–related NCDs among Indians in KwaZulu-Natal (KZN), South Africa. Therefore, the aim of this paper is to compare dietary intakes with the World Health Organization (WHO) population nutrient goals for the prevention of death and disability from NCDs [13] and to describe the dietary quality and patterns in relation to the risk markers for NCDs in an Indian community in KZN, South Africa.

## Methods

### Participants

At the time of the 2011 South African census, the total population of Indians was 1 286 930, of whom the majority, totalling 757 000, resided in the province of KZN [14]. Two-hundred-and-fifty apparently healthy individuals within the age range of 35–55 years were recruited from the homogenous Indian community, Stanger in the province of KZN. Once eligibility was established, participants who were willing to participate in the study were randomly selected. Individuals diagnosed previously with hypercholesterolaemia and/or diabetes mellitus and individuals using dietary food supplements were excluded. The other exclusion criteria were pregnancy, lactation, any acute or chronic illness, use of chronic medication and inability to communicate freely. Informed consent was sought and received from study participants. The study was approved by the Ethics Committee of North-West University (05 M15).

### Questionnaires

Habitual dietary intakes were measured by a validated quantitative food frequency questionnaire (QFFQ) [15] modified to include Indian foods and with a one-month recall period. It was acknowledged that the diet might be affected by seasonal variability. However, fresh fruits and vegetables are widely available throughout the year in KZN while other food products obtained via the retail food markets are not affected by seasonality [16]. In a preliminary study, an inventory of foods commonly consumed by South African Indians was compiled from interviews conducted in 25 randomly selected households. Since the type and amount of ingredients and preparation methods of composite dishes vary according to differences in religious and economic status [17] all traditional recipes were collected. Final construction of the recipes was determined by averaging three recipes per dish. A prototype QFFQ was developed from the food list. Foods were grouped together, starting with the most frequently consumed staple foods and ending with the least common foods, totaling 285 listed foods. Thereafter, the QFFQ was piloted among ten randomly selected households from the preliminary study sample to determine the completeness and appropriateness of the food list.

The QFFQ was then tested for reproducibility and comparative validity against three 24-hour food recalls as the reference method in a sub-sample of 50 participants. The QFFQ, was shown to be relatively reproducible (range for Pearson r: 0.4 to 0.7 – total carbohydrate 0.4, energy 0.44, total fat 0.48, total protein 0.49, total polyunsaturated fatty acids 0.55, niacin 0.63, cholesterol 0.7) but to have moderate to poor validity (range for deattenuated Pearson r: 0.008–0.5 – iron 0.008, energy 0.39, total carbohydrate 0.42, total fat 0.44, total protein 0.46, cholesterol 0.5) in comparison to the reference method. Recommendations derived from the comparative validity and reproducibility results were taken into account in the main study to limit quantification, portion size estimation error and respondent fatigue. The person responsible for food preparation was required to be present at the interview to assist the participant to estimate the amount of foods consumed. Measures were put into place to limit participant fatigue due to the length of the QFFQ by arranging convenient interview times. The first 50 participants completed three 24-hour food recalls and two QFFQs (as part of the comparative reproducibility and validation study); thereafter, the remaining 200 participants completed one QFFQ and 24-hour food recall. In total, 250 participants were interviewed by trained field workers. A random selection of days was used. Weekends and days that were unusual in terms of normal dietary intake – e.g. dry, saltless and whole-food fasting, which are typical of a Hindu’s religious prescription – were avoided. In order to determine portion sizes, the Medical Research Council (MRC) Dietary Assessment Education Kit was used in combination with typical household utensils [18].

### Physical and biochemical measurements

Anthropometric measurements [weight, height and waist circumference (WC)] were measured using standardised techniques and instruments [19]_._ Participants were instructed to fast for 10 h before biochemical assessment. Blood pressure was recorded using a standard mercury sphygmomanometer according to standard guidelines. Only finger-prick blood samples were taken because of limited financial and infrastructure resources. Fasting blood glucose, triglycerides and total cholesterol were recorded using the Accutrend meter (Accutrend GCT kit, Accu-Check Softclix Pro Lancets) and strips (Accutrend Cholesterol, Glucose and Triglyceride Strips) supplied by Roche Products Pty Ltd, Randburg, Gauteng. All biochemical measurements were recorded by a registered nurse.

### Score risk marker

Asian Indians have been shown to have more than a two-fold risk of dying from coronary artery disease (CAD) from any combination of risk factors as compared with Caucasians [20]. For comparative purposes, two scores were developed for this study; namely “risk score”, which was an aggregate total of all risk markers defined by Asian standards [21] and “risk score 1” which was the aggregate of all risk markers, with fasting glucose counting twice as much as the other risk markers because of many and strong correlations with nutrients.

### Dietary data analysis

The FoodFinder 3® dietary analysis software program [22] based on the South African MRC food composition tables was used to capture the dietary data. Indigenous Indian ingredients and recipes not coded in the MRC food composition tables were added, using the nutritive value of Indian foods as a guide [23].

In order to determine the relationship between food consumption and disease prevalence, dietary quality and patterns were determined by index construction and factor analysis. Two dietary quality indices, a deficiency index (Index 1) and an excess index (Index 2), were calculated from the unadjusted nutrient intakes, following the procedure of Thiele *et al.* [24]. Calculation of the deficiency index (a summary score similar to the nutrient adequacy and mean nutrient adequacy ratios [25]) and the excess index provide a summary of both adequacy and the risk of excess of the total diet which might be lost in a single aggregate score [24].

In preparation for factor analysis, food items from the QFFQ were aggregated into 12 food groups based on the system of the International Network of Food Data Systems (INFOODS) [26] using the FoodFinder 3 program® [22]. The percentages of total energy, total fat, saturated (SFA), PUFA, monounsaturated fat (MUFA), n-3 and n-6 fatty acids derived from each food group were calculated.

### Statistical analyses

The Statistical Package for Social Science (SPSS) [27] version 17® was used to calculate frequency tables, means, medians, and standard deviations for anthropometric and biochemical measurements, nutrient intakes and diet quality. Pearson partial correlations, using BoxCox transformed data and controlled for age and smoking were used to detect associations between diet indices and clinical risk markers. Principal component analysis with Varimax rotation was performed on the percentage total fat derived from each of the food groups. Principal components derived from the percentage total fat intake with eigenvalues of >1 were used for further analyses [28]. Factor scores were calculated by adding the percentage fat from each food group weighted by the factor loading for each participant [29], and divided into quintiles of the distribution. Medians of the risk markers were calculated for the first quintile, second to fourth quintiles combined, and fifth quintile. The Jonckheere-Terpstra test [30] was used to test for trends in the medians of the risk markers for the above-mentioned three groups of factor scores.

## Results

### Anthropometric characteristics and clinical profile

The anthropometric characteristics and clinical profile for the whole sample are shown in Table 1. Cut-off points for clinical parameters were defined as follows: impaired fasting blood glucose as >5.55 mmol/L, hypercholesterolaemia as >5.2 mmol/L, hypertriglyceridaemia as >1.69 mmol/L and hypertension as SBP ≥130 or DBP ≥85 mmHg [31]. The majority of participants had diastolic blood pressure measurements above the cut-off value (94 % of men; 92 % of women). Fasting blood glucose levels were higher than the cut-off (>5.55 mmol/L) for 37 % men and 40 % women. Total blood cholesterol of 63 % of men and of 22 % of women was >5.2 mmol/L. Both men (92 %) and women (86 %) recorded raised triglyceride levels (>1.69 mmol/L). Body mass index (BMI) was classified according to the BMI criteria for Asians [21]. WC cut-off points were based on the recommendations of the WHO for Asian populations, defined as ≥90 cm for men and ≥80 cm for women for central obesity [31]. Hence, 86 % of the men and 94 % of the women were classified as centrally obese.

**Table 1:** Anthropometric characteristics and clinical profile with reference to Asian cut- off points for men and women [21, 31]

Variable	Asian cut-offs	Total group	Men	Women
		*N* = 250	*N* = 111	*N* = 139
SBP	<130 mmHg	93.6	93.6	93.5
	>130 mmHg	6.4	6.3	6.4
DBP	<85 mmHg	7.2	6.3	7.9
	>85 mmHg	92.8	93.6	92
Fasting glucose	<5.55 mmol/L	61.2	63	59.7
	>5.55 mmol	38.8	36.9	40.2
Total serum Cholesterol	<5.2 mmol/L	60	36.9	78.4
	>5.2 mmol/L	40	63	21.5
Triglycerides	<1.69 mmol/L	11.2	8.1	13.6
	>1.69 mmol/L	88.8	91.8	86.3
WC (central obesity)	≥90 cm (men)		86	
	≥80 cm (women)			94
BMI (kg/m^2^)	Underweight <18.4	0.4	0.9	0
	Normal 18.5–−22.9	11.6	5.4	16.5
	Overweight 23.0–24.9	28.8	36	23
	Obesity class I 25.0–29.9	44.8	44.1	43.1
	Obesity class II 30.0>	15.6	13.5	17.2

*SBP* systolic blood pressure, *DBP* diastolic blood pressure, *WC* waist circumference, *BMI* body mass index

### Nutrient intake

Table 2 presents the mean percentage energy distribution, cholesterol, sodium, fruit and vegetable and dietary fibre intakes in comparison to the WHO population nutrient intake goals for the prevention of death and disability [13]. The mean percentages of energy from total fat, PUFAs, n-6 fatty acids and free sugars were higher than the WHO goal. However, n-3 fatty acids, dietary fibre and fruit and vegetable intakes were lower than the recommended goal.

**Table 2:** Comparison of mean intakes of men and women with the WHO Population nutrient intake goals for prevention of death and disability from NCDs [13]

Food/nutrient	Goal	Mean reported intakes	
		Men	Women
		*N* = 111	*N* = 139
Total fat %E	15–30	35.1	37.1
SAT FAT %E	<10	10.0	9.4
PUFAs %E	6–10	12.4	11.2
n-6 PUFAs %E	5–8	11.7	10.3
n-3 PUFAs %E	1–2	0.25	0.23
Trans fatty acids %E	<1	0.96	0.86
MUFAs %E	By difference^a^	9.6	8.9
Total carbohydrate %E	55–75	49.9	47.0
Free sugars %E	<10	12.5	12.5
Protein %E	10–15	12.8	12.0
Cholesterol mg/day	<300	204.7	184.3
Sodium mg/day	<2000	1899	1739
Fruit and vegetables g/day	≥400	221.9	236.9
Dietary fibre g/day	>25	18.8	18.1

*%E* percentage of energy intake, *SAT FAT* saturated fatty acids, *PUFAs* polyunsaturated fatty acids, *MUFAs* monounsaturated fatty acids

^a^Calculated as total fat - (saturated fatty acids + polyunsaturated fatty acids + trans fatty acids)

### Diet quality

Overall diet quality indices combine many components of the diet into a single indicator. For the deficiency and excess indices, the higher the index, the better the diet quality. In Table 3, the means for the deficient index (Index 1) reflect a moderate to good diet quality whereas the excess index (Index 2) reflects good diet quality. In terms of nutrient adequacy (deficient index), there were significant positive partial correlations (corrected for age and smoking) for risk score, risk score 1 and blood glucose levels for men and women, waist circumference for men and for blood cholesterol levels for women (Table 4) The excess index (Index 2) was significant and inversely correlated with risk score and risk score 1 for men, but showed weak, non-significant positive partial correlations for women. For men, the excess index was negatively correlated with all clinical risk markers, with the partial correlations with waist circumference and blood glucose levels being significant (*p* < 0.05). Similarly to the risk scores, partial correlations of clinical risk markers with the excess index were weak and positive.

**Table 3:** Mean (SD) and median (25^th^, 75^th^) dietary quality indices scores

Indices	Total group *N* = 250			Men *N* = 111			Women *N* = 139		
	Mean (SD)^a^	Median	25^th^, 75^th^ percentile	Mean (SD)^a^	Median	25^th^, 75^th^ percentile	Mean (SD)^a^	Median	25^th^, 75^th^ percentile
^b^Index 1	2259.8	2260	2143	2255	2247	2129	2265	2274	2156
	(198.4)		2410	(204.7)		2418	(187.2)		2410
^c^Index 2	518	522	502.5	515.1	516	502.9	522.1	526	501.9
	(35.4)		540.5	(34.1)		534.3	(35.5)		548.5

^a^ *SD* standard deviation

^b^Deficiency index, max score 3000

^c^Excess index, max score 600

**Table 4:** Pearson partial correlations* controlled for age and smoking between risk scores, clinical factors and diet index scores

	Index	^a^Risk Score	^b^Risk Score 1	WC	SBP	DBP	Glucose	Chol	Trig	BMI
*N* = 250	INDEX 1	0.21	0.2	0.12	0.01	0.01	0.24*	0.11	−0.02	0.06
	INDEX 2	−0.10	−0.1	−0.13*	0.01	0.01	−0.06	−0.01	0.04	−0.00
Men *N* = 111	INDEX 1	0.23*	0.2*	0.21*	−0.06	−0.03	0.26*	−0.05	−0.02	0.10
	INDEX 2	−0.30*	−0.2*	−0.28*	−0.06	−0.02	−0.22*	−0.12	−0.14	−0.17
Women *N* = 139	INDEX 1	0.23*	0.2*	0.14	0.06	0.05	0.22*	0.24*	−0.01	0.04
	INDEX 2	0.04	0.0	0.05	0.06	0.02	0.03	0.07	0.13	0.08

*WC* waist circumference, *SBP* systolic blood pressure, *DBP* diastolic blood pressure, *BMI* body mass index, *Chol* cholesterol, *Trig* triglyceride, *Index 1* deficiency index, max score 3000, *Index 2* excess index, max score 600

^*^Pearson correlation:*P < 0.05

^a^Risk score is the aggregate total of all risk markers defined by Asian standards

^b^Risk score 1 is the aggregate of all risk markers, but fasting glucose was counted twice

### Dietary patterns

Two dietary patterns were identified with eigenvalues of >1. Table 5 presents the factor loadings for each pattern. Factor 1 accounted for 20.2 % and factor 2, 17.7 % of the total variance. Factor loadings were interpreted as Pearson correlation coefficients, where each factor loading is the correlation of the food group with the dietary pattern [28]. Factor 1 was characterized by strong negative correlations for the meat and meat products (−0.88), fish and seafood (−0.64) groups and strong positive correlations for the legume (0.75) and cereals and cereal products (0.61) groups. Factor 2 had a strong negative loading for the nuts and seeds group (−0.70), contrasted with strong positive loadings for the sugar and sweets (0.55) and visible fats and oil (0.51) groups. Since Factor 1 appeared to contrast the meat and meat products and fish and seafood groups (strongest negative factor loadings) with the legumes and cereals groups (strongest positive loadings) it was named the “meat and fish versus legume and cereal pattern”. Factor 2 was identified as the “nuts and seeds versus sugars and fat pattern”. Based on the factor loadings, the lowest quintile for the meat and fish versus the legume and cereal pattern represents participants with higher than the sample average meat, meat products and fish intakes, while participants in the highest quintile were those with above average intakes of legumes and cereals. Likewise, for the nuts and seeds versus sugars and fat pattern, factor scores in the first quintile were associated with higher than the sample average nut and seed group intake, contrasted with higher than average intakes of the sugars and visible fats groups in quintile 5. Table 6 shows the medians of the clinical risk markers for the first, second to fourth combined, and fifth quintiles of factor scores. The medians for WC and blood glucose, cholesterol and triglyceride levels showed significant decreasing trends from Quintile 1 (participants with higher than the sample average intake of the meat and fish group) to Quintile 5 (participants with higher than the sample average of the legume and cereal group) for Factor 1 (*p* < 0.05). For Factor 2, the medians for all risk markers, except WC, decreased from Quintile 1 (participants with higher than the sample average intake of the nut and seed group) to Quintile 5 (participants with higher than the sample average intake of the sugars and visible fat group), however, only the trends for blood glucose and cholesterol were significant (*p* < 0.01).

**Table 5:** Factor loadings for the first two principal components identified from the percentage total fat contributed by each food group

Food group	Factor loadings	
	Factor 1: Meat and fish versus legume and cereal pattern	Factor 2: Nuts and seeds versus sugars and fat pattern
Cereals, cereal products	0.61	−0.04
Vegetables	0.55	−0.29
Fruit	0.08	0.30
Legumes	0.75	−0.01
Nuts and seed	0.06	−0.70
Full-fat milk, milk products	0.24	0.37
Low-fat milk, milk products	0.13	0.34
Eggs	−0.54	0.03
Meat and meat products	−0.88	0.06
Fish and seafood	−0.64	−0.40
Visible fats and oils	−0.01	0.51
Sugar and sweets	0.17	0.55

**Table 6:** Median values of risk markers according to the 3 groups of quintiles for factor 1 and factor 2 scores

Risk marker	Factor 1: Meat and fish versus legume and cereal pattern				Factor 2: Nuts and seeds versus sugars and fat pattern			
	Q1	Q2-Q4	Q5	P for trend^1^	Q1	Q2-Q4	Q5	P for trend^1^
SBP (mmHg)	121.0	122.0	120.0	0.499	125.0	122.0	120.0	0.097
DBP (mmHg)	90.0	90.0	90.0	0.753	90.0	90.0	90.0	0.378
WC (cm)	92.0	88.5	87.0	0.015*	86.2	90.0	88.0	0.129
Blood glucose (mmol/L)	5.5	5.3	5.1	0.008*	5.5	5.3	5.1	0.003**
BMI (kg/m^2^)	25.2	25.4	25.5	0.542	26.1	25.4	24.7	0.086
Blood Cholesterol (mmol/L)	5.0	4.9	4.7	0.004*	5.1	4.9	4.8	0.008**
Blood Triglyceride (mmol/L)	2.1	2.1	2.0	0.040*	2.2	2.1	2.0	0.187

*SBP* systolic blood pressure, *DBP* diastolic blood pressure, *WC* waist circumference, *BMI* body mass index

^1^Jonckheere-Terpstra test:*P < 0.05,**P < 0.01

## Discussion

The role of diet in the aetiology of most NCDs is extremely important. A plethora of scientific publications has revealed – through ecological and epidemiological studies, interventions with specific nutrients and foods in placebo-controlled trials and through molecular and genetic research – that there is a consistent relationship between unhealthy diets and the emergence of NCDs [2], making nutrition a major modifiable determinant of NCDs. The investigation of diet quality and patterns in relationship to the high prevalence of NCDs in this population paves the way for ethno-sensitive prudent intervention.

Using Asian cut-offs, the assessment of anthropometric characteristics and clinical profile led to the identification of a number of risk markers for NCDs, which supports the current high prevalence of NCDs in the Indian community [2, 3]. Although HDL-C levels were not measured, the results of the anthropometric and clinical measurements suggest that the participants displayed characteristics typical of the metabolic syndrome that is common among Asian Indians [32].

The comparison of food and nutrient mean intakes with the WHO population nutrient goals for the prevention of death and disability from NCDs [13] showed that the mean percentage energy from total fat, free sugars and n-6 fatty acids were above the recommended goals. Similar percentages of energy from total fat have been reported from Indians living in the metropolitan area of Durban, [33] and from Asian Indian migrant studies, [34, 35] reaffirming the nutrition transition [1]. We found that the mean percentage energy from n-3 fatty acids and from carbohydrate, and the mean daily intakes of fruit and vegetables and fibre were below the recommended WHO goals. The mean fruit and vegetable intake of the total sample in the current study was only slightly over half the recommended WHO intake [13]. A study on the association of fruit and vegetable intake with CAD in urban South Indians reported slightly higher mean intakes (260 g/day and 270 g/day for men and women respectively) [36]. Although Indians in South Africa consume a variety of fruit and vegetables in their diet, the apparent low intake of fruit and vegetables could be because vegetables are often added to a meat dish as an extender and for this reason, are classified under the meat group, masking the actual intake of vegetables.

In agreement with Wolmarans *et al.* [33], our results showed PUFAs provided more than 10 % energy. In this study, the mean percentage energy derived from n-6 fatty acids (11.7 % for men and 10.3 % for women) exceeded the recommended WHO goal of 5 to 8 %. In contrast, the percentage energy derived from n-3 fatty acids (0.25 % for men and 0.23 % for women) was lower than the WHO recommendation of 1 to 2 %, reflecting the Western diet trend. Expressing the intake of PUFAs as a mass is considered a better approach to n-6 and n-3 fatty acid balance than a ratio [37]. The United Kingdom (UK) Food Standards Agency Workshop [38] recommended that the use of the ratio to estimate CVD risk should be abandoned. South Asians have been reported to consume significantly less n-3 fatty acids (0.08 %E *versus* 0.13 %E) but more n-6 fatty acids (5.4 %E *versus* 5.0 %E) than Caucasians in the UK [37]. The n-3 fatty acid intake was possibly low in the current study because most sources rich in n-3 fatty acids do not feature in the South African Indian diet for several reasons. Oily fish intake is largely restricted because of lack of availability and high cost. Sunflower oil is the preferred cooking oil in terms of cost, taste and availability.

The percentage energy from carbohydrates (47.2 % for men and 45.4 % for women) was below the 55–75 % range as recommended by the WHO goals [13]. The reported range for percentage energy from carbohydrates by Wolmarans *et al.* [33], on the dietary intake of Indians living in Durban was similar to our study for men and slightly higher for women. In contrast to the lower percentage energy from carbohydrates, the sugar intake in the current study (12.5 E% for both men and women) was higher than the WHO goals, mirroring similar results by Wolmarans *et al*. [33] with effects noted in the high prevalence of diabetes in this community [3].

With reference to the low fibre intake in this study, it is well recognised that low glycaemic index diets are associated with improved weight loss, decreased fasting glucose and triglyceride levels and lowering of blood pressure [39]. Increased intake of legumes and pulses may contribute to lowering the glycaemic index of the South African Indian diet provided that the cooking methods are appropriate. The American Dietetic Association acknowledges that the consumption of viscous dietary fibre lowers blood cholesterol levels and helps to normalise blood glucose and insulin levels and therefore, is ideal for the treatment and prevention of obesity [40]. Refined carbohydrates such as rice, bread and roti are staple foods for Indians in South Africa and may predispose individuals to atherogenic dyslipidaemia including hyper-triglyceridaemia [41]. In light of this, it is recommended that refined carbohydrates be reduced to achieve optimum triglyceride levels in our study population.

The percentage energy from protein (12.8 % for men and 12.0 % for women) fell within the range of WHO goals. A possible reason explaining the prudent protein intake could be that animal protein intake is limited to certain days of the week because of religious worship, in the form of abstinence from meat. On days of abstinence from meat, the protein intake is balanced by the consumption of legumes (11 %E).

Diet quality and patterns can be used to assess associations between clinical markers and dietary behaviours. The deficient index (mean 2260 out of a possible 3000) and excess index (mean 518 out of 600) calculated in our study reflected moderate to good diet quality. The higher the risk score and risk score 1, the greater the risk towards CAD whereas a high index indicates a better quality of diet. Thus, an inverse correlation could be expected between the values of the risk markers and diet index. The Pearson partial correlation coefficients between the deficient index and risk markers were weak, with only the coefficients for glucose for the whole sample, men and women and waist circumference for the men giving significant positive correlations. The deficient index reflects the adequacy of micronutrients, fibre, protein, carbohydrate and essential fatty acid intakes in comparison to the Recommended Dietary Allowances [25]. Thus, a diet with a high deficiency index score, while of good quality in terms of nutrient adequacy may have characteristics associated with dietary risk of NCDs as seen by the positive correlation with waist circumference and glucose for men (Table 4). The excess index is a measure of how closely the diet meets the prudent diet recommendations and was inversely correlated with WC for the whole sample and for men and with glucose for men, which could be expected: the better the quality of the diet the lower the waist circumference and blood glucose levels.

Two factors were identified from the percentage fat contributed by the food groups. The meat and fish versus legumes and cereal pattern contrasted in Factor 1 in two ways: one in which fat intake from meat, meat products, fish and shellfish was higher than average, and one in which the main contributors to fat intake were legumes, cereal and cereal products and vegetables. The significant decreasing trends in the medians of WC and blood glucose, cholesterol and triglycerides from Quintile 1 to Quintile 5 suggest that a dietary pattern in which a higher proportion of fat is derived from the cereal, legume and vegetable food groups is associated with lower levels of these risk markers. However, since the Quintile 5 medians for WC and blood triglycerides remained above the risk cut-off values, whilst those for blood glucose and cholesterol were only slightly lower than the cut-off values, and the remaining risk markers (SBP, DBP and BMI) were unchanged across quintiles, the cereal and legume pattern cannot be considered as protective against NCD risk. The high loadings for the cereal, vegetable and legume groups obtained in our study can be explained by the traditional food preparation practices of South African Indian cuisine. The fat content in cereals and cereal products and in legume and vegetable dishes is derived from “added” fat, used during preparation and cooking of these foods. For example, onions and spices may be tempered in butter or ghee and tossed into boiled rice or legumes; savoury pastries are fried in sunflower oil; and sweets made from cereals are cooked in a ghee base.

Dietary patterns vary by ethnicity and may change to a new environment [42]. In an Indian Health study across three regions in India, the diet was characterized by dairy, fried snacks and sweets which were positively associated to abdominal obesity [43]. It was also found that there was a continued preference for Indian sweets prepared with sugar and substantial amounts of saturated fat from ghee and butter [43]. Furthermore, the cooking method of vegetables might alter some of their preventative properties due to added fat [44].

The nuts and seeds versus sugars and fats pattern reflects a food pattern with high intakes of inherent fats either in the form of nuts and seeds or as discretionary fat added to foods or in combination with sugars and sweets. Generally, studies examining the dietary patterns frequently choose to name the fruit and vegetable and whole grain loading, the “healthy/ prudent” pattern [45] whereas, dietary patterns characterized by higher intake of fat, meat and refined grain are referred to as the “Western pattern” [46]. The dietary patterns derived from the percentage fat supplied by food groups in our study suggest that a high legume, cereal and cereal products and vegetable dietary pattern may not be a healthier’ dietary pattern versus the meat the meat and fish pattern for Indians in KZN, and could be identified as a “added” fat pattern. The nuts and seeds versus sugar and fat pattern may be the “healthier” pattern in this community as fat content comes from obvious sources, which may be easier to control. As pointed out by several authors the interpretation the results of factor analyses must be done with caution, as factor analyses may be influenced by several subjective decisions, [28, 47, 48] including: whether foods are classified into food groups, the food groups’ used and how foods are classified into food groups, the dietary component analysed, and the selection of factors included in the analyses.

## Conclusions

The overwhelming burden of the nutrition transition in the Indian community is characterised by a shortfall of fruit and vegetable, fibre and n-3 fatty acid intake and the traditional use of added fat in food preparation. We based the factor analysis on the percentage fat intake derived from each food group, as fat intake was the primary focus of our study. Use of percentage energy intake, and/or different food groupings may have resulted in the emergence of different food patterns. Further research is needed for a better understanding of the diet as modifiable risk factor. However, the assessment of diet quality and patterns in this populace, allows for the translation of dietary goals into practical recommendations such as increasing fruit and vegetable, fibre and n-3 fatty acid intake, changing cooking methods, and limiting the use of added fats for a healthier lifestyle.
